# From Hospital to Community: Perspectives on Forensic Pathways for Adults With Intellectual Disabilities

**DOI:** 10.1111/jar.70329

**Published:** 2026-11-02

**Authors:** Alina Haines‐Delmont, Kathryn Fradley, Faye McLoughlin, Sahrish Ali, Sana Raja, Michaela Thomson

**Affiliations:** ^1^ School of Nursing and Public Health Manchester Metropolitan University Manchester UK; ^2^ Calderdale and Huddersfield NHS Foundation Trust Huddersfield UK; ^3^ Mersey Care NHS Foundation Trust Liverpool UK

**Keywords:** care pathways, continuity of care, discharge planning, forensic mental health, intellectual disabilities, qualitative research

## Abstract

**Background:**

Adults with intellectual disabilities and forensic histories often experience prolonged hospital stays and delayed discharge, yet evidence on how forensic pathways support safe and timely transition to the community remains limited.

**Method:**

A qualitative, multi‐perspective study was conducted within a large healthcare organisation in England. Semi‐structured interviews and focus groups were undertaken with service users (*n* = 11), family members/carers (*n* = 5), and multidisciplinary staff (*n* = 18), alongside two co‐production workshops with 92 professionals. Data were analysed using reflexive thematic analysis.

**Results:**

Discharge was experienced as a cyclical and unstable process rather than a linear event. Three interrelated themes emerged: (1) establishing readiness and foundations for continuity, (2) maintaining coordination and coherence across the care trajectory, and (3) sustaining community living while preventing re‐entry into institutional care.

**Conclusions:**

Supporting safe and sustainable discharge requires continuity‐focused integrated pathways beginning at admission and extending beyond discharge to reduce repeated cycles of admission.

## Lay Summary

- People with intellectual disabilities who are involved with forensic mental health services often experience long hospital stays and repeated admissions because discharge is not well supported.
- This study shows that discharge is not a single event, but part of an ongoing journey that needs consistent support before, during, and after leaving hospital.
- Listening to people with lived experience, families, and staff highlights the importance of trust, clear and early planning, and strong community support to help people stay well outside hospital.
- The findings can help services and commissioners improve forensic pathways and reduce unnecessary time spent in institutional care, additional trauma, and deterioration of health for service users and extensive costs associated with forensic services.

## 1 Introduction

The care and treatment of adults with intellectual disabilities (‘learning disabilities’ in the UK) and co‐occurring mental health problems who are detained under mental health legislation in England and Wales (Department of Health 2015) (as amended by the Mental Health Act 2025) represent a persistent and complex challenge for health and social care systems. People with intellectual disabilities in forensic services may experience prolonged inpatient stays, delayed discharge, and inconsistent access to appropriate community support (McKenna et al. 2025).

The exposure of institutional abuse at Winterbourne View Hospital in 2011 marked a turning point, although subsequent events at Whorlton Hall and Cawston Park demonstrated that serious institutional failings persisted (Department of Health 2012; British Broadcasting Corporation 2019; NHS England 2021). The national *Transforming Care* programme and subsequent *Building the Right Support* strategy sought to reduce long‐stay inpatient provision and expand community‐based support, but progress has remained uneven (NHS England 2015, 2023b).

Despite longstanding efforts to reduce inpatient provision, 2130 people with intellectual disabilities and autistic people were recorded in inpatient services in England at the end of May 2026; 1030 (49%) had a total length of stay exceeding 2 years (NHS England Digital 2026). This population includes adults detained under Part III of the Mental Health Act 1983 in England and Wales following criminal proceedings, some of whom are subject to Ministry of Justice restrictions affecting leave and discharge (Department of Health 2015). For this group, treatment, public protection, legal restrictions and community resettlement must be addressed concurrently. Limited community provision, fragmented inter‐agency coordination and risk‐averse decision‐making can contribute to delayed discharge (Ince et al. 2022; Taylor et al. 2017), while prolonged hospitalisation may restrict autonomy, relationships and community participation and generate substantial costs for health and social care systems (Killaspy et al. 2016; Sá‐Fernandes et al. 2025; Saini et al. 2025). Recent policy in England therefore continues to emphasise reducing reliance on inpatient care, strengthening community provision, addressing health inequalities and improving workforce knowledge and skills (NHS England 2019, 2024, 2025a).

Despite successive policy commitments, evidence concerning how forensic pathways should be organised to support safe, effective and timely discharge remains limited. A recent scoping review identified few international studies describing structured models of forensic care for adults with intellectual disabilities, limited evaluation of their effectiveness or implementation, and under‐representation of the perspectives of people with lived experience and families (Haines‐Delmont et al. 2025). International policy similarly supports a move towards community‐based, person‐centred and rights‐respecting mental health care: the World Health Organisation advocates community‐based mental health systems, while the United Nations Convention on the Rights of Persons with Disabilities (UN CRPD) affirms autonomy, inclusion and the right to live in the community (United Nations 2006; World Health Organization 2025a, 2025b). Although national policy frequently considers people with intellectual disabilities and autistic people together, the present study focused specifically on adults whose primary diagnosis was an intellectual disability.

This study aimed to explore service‐user, family/carer, and multidisciplinary staff experiences of discharge and community transition for adults with intellectual disabilities and forensic histories, in order to identify practice‐based recommendations for improving forensic pathways.

## 2 Methods

### 2.1 Design and Ethical Approval

This qualitative study adopted a multi‐perspective design involving service users, clinical staff, and family members of adults with intellectual disabilities and forensic histories. Data were collected through semi‐structured face‐to‐face interviews and hybrid focus groups, in which participants could join either face‐to‐face or online via Microsoft Teams, followed by face‐to‐face co‐production workshops to validate and refine findings and identify key recommendations for change. The study sought to understand discharge as experienced across the forensic care trajectory by integrating perspectives from service users, family members, and multidisciplinary professionals working across inpatient and community forensic intellectual disability services within a large mental health trust in England.

Ethical approval was obtained from the Yorkshire & The Humber ‐ Leeds West Research Ethics Committee (REC) (23/YH/0273), Health Research Authority (HRA) (IRAS project ID: 324747) and the Research and Governance Team at Manchester Metropolitan University (EthOS Reference Number: 49315).

Local Research and Development governance permissions were granted. All participants provided written informed consent and were assured of confidentiality and the voluntary nature of participation. To protect anonymity, each participant was assigned a unique code.

### 2.2 Participants and Recruitment

A purposive sampling strategy was used to recruit participants with direct experience of forensic intellectual disability services, including people currently detained under the Mental Health Act 1983 in England and Wales and those discharged into community settings. Recruitment took place within a large NHS mental health trust in England and was facilitated through clinical teams and site champions within participating services. Potential participants were provided with accessible information sheets explaining the study aims, procedures, and safeguarding arrangements. Potential participants had the opportunity to discuss the study with a member of the research team before deciding whether to participate and provided written informed consent prior to data collection.

A total of 34 participants took part in the study:

- Service users (*n* = 11): Adults with a primary diagnosis of intellectual disability (ICD‐11) who were detained under civil provisions, including Section 3, or forensic provisions, including Sections 35, 37, 41, 47 and 49, of the Mental Health Act 1983 in England and Wales. Participants had varied co‐occurring mental health and personality‐related diagnoses, alongside histories of offending behaviour. The study focused on adults whose primary diagnosis was an intellectual disability; autism was not an inclusion criterion and was not examined as a distinct analytic characteristic.
- Family members/carers (*n* = 5): Relatives or primary carers of adults currently or previously detained within forensic intellectual disability services.
- Clinical staff (*n* = 18): A professionally diverse multidisciplinary group working across inpatient and community forensic intellectual disability services, comprising nursing professionals (*n* = 6), psychology professionals (*n* = 4), allied health and social care professionals (*n* = 6), and forensic psychiatrists (*n* = 2).

### 2.3 Data Collection

Data were collected between March 2024 and May 2025 through five focus groups and 14 semi‐structured interviews, conducted by members of the research team [MT, SA, AHD, SR]. Some interviewers had pre‐existing professional relationships with participants, while others were independent researchers to reduce potential bias. All participants were informed of the study's aims and procedures prior to taking part.

- Focus groups: Conducted in a hybrid format (face to face and online, via Microsoft Teams), audio‐recorded with consent. Participants could choose to disable their cameras if preferred.
- Interviews: Conducted face‐to‐face. For service users and family members, interviews were facilitated by experienced qualitative researchers and supported by practitioners to ensure participants' wellbeing and debriefing after sessions.

Two co‐design workshops were conducted in September 2024 and April 2025 with a total of 92 attendees (*n* = 27 at the first workshop and 65 at the second). The workshops were used to validate, refine, and prioritise emerging findings and to co‐develop feasible recommendations for improving forensic discharge pathways.

Interview and focus group guides were developed collaboratively by the research team, drawing on previous patient and public involvement and engagement, stakeholder consultation and findings from the preceding scoping review (Haines‐Delmont et al. 2025). Guides were tailored to participant type and explored experiences of admission, treatment, discharge planning, and transition to the community.

### 2.4 Data Analysis

All interviews and focus groups were audio‐recorded and transcribed verbatim. Transcripts were uploaded into NVivo 14 (Lumivero 2023) for management and coding and analysed using reflexive thematic analysis (Braun and Clarke 2021; Braun et al. 2023). This enabled an inductive exploration of patterns across participant groups while remaining attentive to contextual and organisational influences. The analytic process involved multiple stages: familiarisation with the data, initial coding, development of preliminary themes through clustering of codes, and refinement into overarching themes. Peer debriefing was conducted for all preliminary themes, and further refinement occurred through multiple team discussions to enhance the rigour of the analysis.

Although the same analytic framework was applied across datasets, the analytic procedures varied between the staff dataset and the service‐user and family datasets. Staff transcripts were independently analysed by four members of the research team (FM, AHD, MT and SA), and the resulting themes were subsequently validated through a co‐design workshop held in September 2024 and included 27 attendees. Service user and family accounts were analysed by five researchers (AHD, MT, SA, SR and KF). Preliminary findings for service users and family accounts were presented for validation and refinement at the second workshop in April 2025, during which feasible recommendations for improving the discharge pathway were also co‐developed.

Initially, interviews and focus groups from each participant group were analysed separately to develop preliminary themes. Themes were subsequently compared across service‐user, family and staff datasets through an iterative process of triangulation. Areas of convergence, divergence and complementarity were examined collaboratively to develop an integrated interpretation of the forensic care trajectory across stakeholder perspectives.

To enhance trustworthiness, the team used reflexive memos and engaged in peer‐debriefing throughout the analytic process. Divergent perspectives were discussed collaboratively and used to question, deepen, and refine the analysis. Recruitment ceased once the planned sample had been achieved and sufficient depth and diversity of perspectives had been generated to address the research aim.

### 2.5 Trustworthiness and Reflexivity

The research team comprised professionals from psychology and health services research, including members with clinical and academic experience in forensic intellectual disability services. The analysis was informed by an interpretivist qualitative approach, recognising that themes were developed through researchers' active engagement with participants' accounts and the data. Reflexive discussions were held throughout data collection and analysis to consider how researchers' professional backgrounds, assumptions and relationships with the study setting may have shaped data generation and interpretation. Reflexive memo‐ing, peer debriefing and collaborative interpretation supported transparency and analytic rigour.

## 3 Results

Three interrelated themes were developed from the analysis: (1) *establishing readiness and foundations for continuity*; (2) *maintaining coordination and coherence across the care trajectory*; and (3) *sustaining community living while preventing re‐entry into institutional care*. Together, the themes represent participants' accounts of admission, inpatient care, discharge planning, community transition and, for some, re‐admission. Figure 1 presents the themes and associated sub‐themes across the forensic care trajectory. Illustrative quotations are presented throughout, with participant identifiers indicating service users, staff, or family members/carers.

**FIGURE 1:**
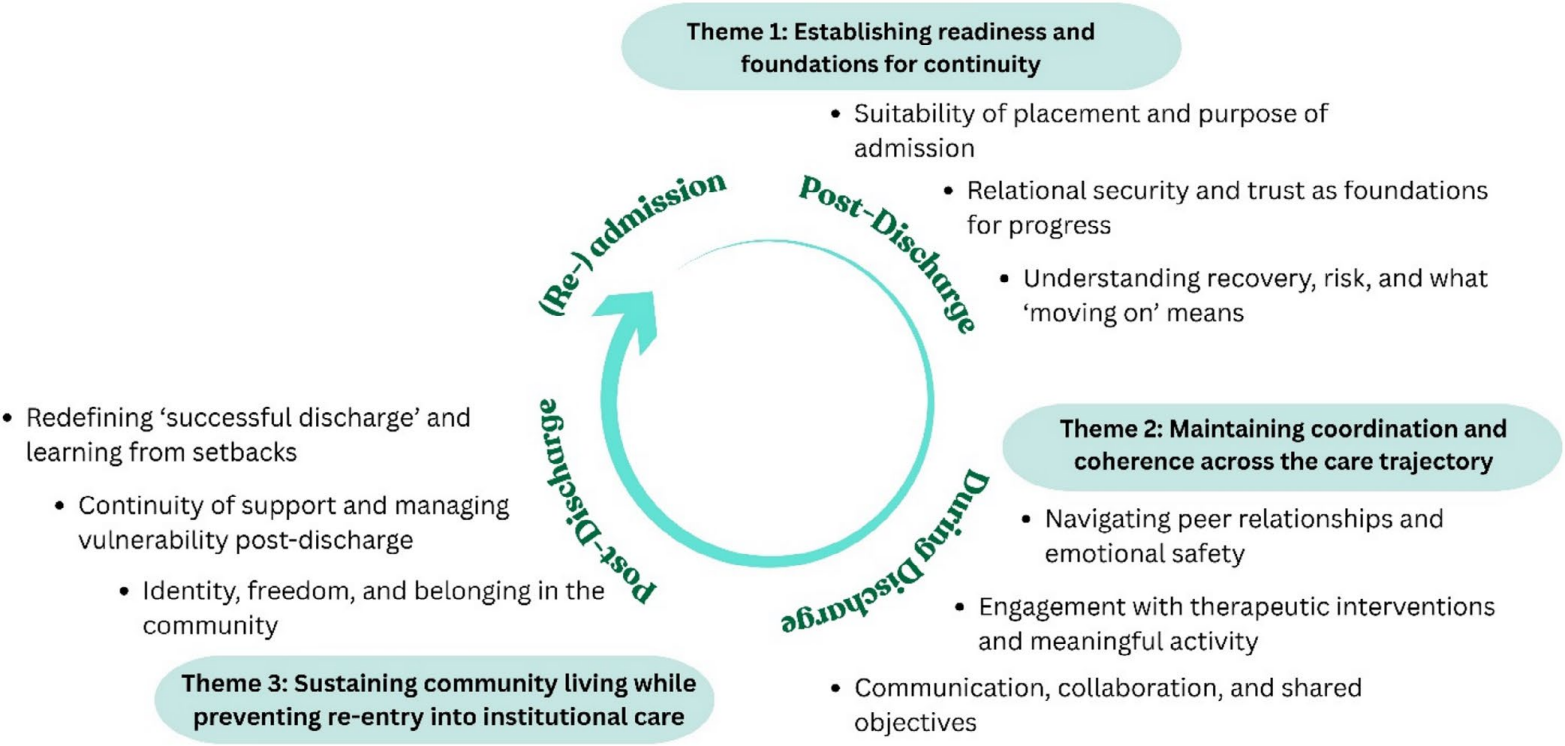
Themes and sub‐themes across the forensic care trajectory.

### 3.1 Theme 1. Establishing Readiness and Foundations for Continuity

Across participant groups, readiness for discharge was described not as a discrete milestone reached at the end of an inpatient stay, but as a process beginning early in the care trajectory and shaped by the quality of preparation, relationships, and shared understanding developed over time. Service users, staff, and family members consistently emphasised that when these foundations were unclear or fragmented, progress stalled and individuals became vulnerable to prolonged stays or repeated cycles of admission and discharge.

#### 3.1.1 Sub‐Theme 1.1 Suitability of Placement and Purpose of Admission

Participants across all groups highlighted the importance of admission having a clear purpose and being matched to the individual's needs, risks, and stage of recovery. Staff described situations in which individuals remained in secure services despite being clinically ready to progress. Reported barriers included limited availability of suitable community placements or systemic barriers beyond the control of inpatient teams, for example unsuitable staffing due to issues with recruitment and retention; pressures on providing placement to cover for admission, discharge and movement within the pathway (between prison, high—medium‐ low‐ secure services); over or under‐stimulating environment; institutional ‘blanket’ rules and repeated or prescriptive assessments.

One staff member stated ‘I never feel confident that that placement's going to support someone’ (Staff focus group #3, P1), while another reflected:

[…] if we're trying to move people on, moving people to community, the challenges of that because of placements and readiness for that and then we get stuck in those cycles of people being stuck within our system when actually from our team perspective, they are very much ready and we've done what we can do. But then what we have is we maybe have people who are sat within our system who unfortunately, as a consequence of being so long sat in our system, that then has a detrimental effect and our pathway of what we see where they should be going or what we could be doing, then gets disrupted. So we end up dealing with other issues.

(Staff focus group #5, P7)

Service users similarly described being moved between services without a clear rationale, often linked to changing diagnoses or shifting interpretations of need. One participant expressed frustration at repeated reassessment and uncertainty about where they belonged within the system:

[…] It's just finding the right placement for me because I'm passed from pillar to post. […] From pillar to post of diagnoses. Now, I've come here, mental health I am now. […] When I came in it was LD now they're saying I don't have LD.

(Service user interview #6, Inpatient)

Family members echoed these concerns, expressing a lack of confidence that placements could meet their relative's needs over time. One carer described their anxiety about the limitations of community provision:

The person from outside was coming in saying well we won't be able to set anything up like this that he needs, look at his needs. Look at how massive they are. We can't do that. We can't provide that sort of level of care with that sort of backing.

(Carers couple interview #1, P1)

Collectively, these accounts suggest that the suitability of placement is not merely a logistical decision but a foundational factor influencing continuity across the care trajectory. When placements lacked a clear purpose or were poorly matched, participants described early erosion of hope and confidence in progression.

#### 3.1.2 Sub‐Theme 1.2. Relational Security and Trust as Foundations for Progress

Relational security emerged as central to establishing readiness and sustaining progress across the pathway. Service users consistently emphasised the importance of feeling safe, understood, and supported by staff they trusted. One service user described how consistent, compassionate relationships helped them cope during periods of distress:

I was emotional and crying and upset and, obviously, I was quite suicidal. […] But [Staff member] used to sit down to me and say, “Look, you don't need to be scared. […] You're not going to be hurt; you're not going to be bullied. You're going to be looked after.”

(Service user interview #4, Community).

Staff similarly highlighted the importance of relational continuity, noting that long‐standing relationships enabled better understanding of individuals' needs and risks:

…there was a lot of trust, a lot of staff that had been there for a long time and they knew the patients.

(Staff focus group #1, P3).

Carers reinforced the value of relational security, particularly for individuals with histories of trauma or mistrust or for individuals with long‐term or repeated admissions:

…they worked out ways to keep him safe […] he learnt to trust them because that was a big thing for him.

(Carers couple interview #1, P2)

However, staff also described how workforce instability and service pressures often undermined these relationships, disrupting continuity and delaying progress. High staff turnover and inconsistent staffing were perceived to limit staff's knowledge and understanding about patients and their needs, which in turn led to breakdowns, escalations in behaviour and inappropriate use of force, restriction and ultimately regression within the pathway.

Participants across groups suggested that without stable, trusting relationships, therapeutic engagement and confidence in discharge planning were significantly compromised.

#### 3.1.3 Sub‐Theme 1.3. Understanding Recovery, Risk, and What ‘Moving on’ Means

A further element of readiness concerned shared understanding of recovery, risk, and the criteria for progression. Service users frequently described uncertainty about what they needed to achieve to move on, contributing to frustration and feelings of powerlessness. Motivation to progress was often framed in behavioural terms, linked to perceived expectations of compliance:

So, I had something to look forward to. It kept me motivated to say, “Oh, you can't do nothing. You've got to behave yourself so you can get out”.

(Service user interview #2, Community).

Others articulated aspirations for independence and control over their own lives:

I said to them once I want to be independent, I want to get my own flat and be independent, you know, at my own pace.

(Service user interview #4, Community).

Service users' accounts conveyed intrinsic motivation and self‐determination, a deep sense of purpose and agency, with progress understood as depending both on external support and their own efforts to change. Independence was described as both a goal and a symbol of recovery, represented as freedom, responsibility, and reconnection with life beyond hospital walls. For many, engaging with opportunities to make choices, demonstrate capability, and access the community fostered a growing sense of autonomy and self‐efficacy.

Staff recognised the challenges of balancing hope with uncertainty, particularly when discharge decisions were influenced by external agencies and legal restrictions. Confusion about ongoing restrictions following discharge was a recurring issue for service users, who described risk‐averse practices continuing into the community without clear explanation:

I said, why do I need staff with me 24 hours?

(Service user interview #1, Community).

So it's just stupid that they're making slight adjustments to the house, when at the end of the day, it's me that's going to be living here. […] Eventually I'm not going to be under his care […] but the doctor at the other hospital is […] still saying, oh, you can't go out after a certain time.

(Service user interview #3, Community)

Carers similarly reported difficulties understanding discharge processes and ongoing restrictions, reinforcing the need for transparent, consistent communication across settings.

Taken together, these findings demonstrate that readiness for discharge is a relational and systemic process that begins early in the care trajectory and evolves over time. Suitability of placement, relational security, and shared understanding of recovery and risk were interdependent foundations shaping whether continuity across the forensic intellectual disability care trajectory could be established. When these foundations were weak or disrupted, participants described stalled progress, diminished hope, and increased vulnerability to prolonged detention or repeated cycles of admission and discharge.

### 3.2 Theme 2. Maintaining Coordination and Coherence Across the Care Trajectory

While establishing readiness provided the foundations for continuity, participants consistently emphasised that sustaining progress depended on how effectively care was coordinated and experienced across the inpatient pathway. Service users, staff, and carers described how everyday interactions, therapeutic engagement, peer dynamics, and planning processes either maintained momentum or contributed to fragmentation and stagnation. This theme captures the mechanisms through which continuity was either strengthened or undermined during individuals' time in secure services.

#### 3.2.1 Sub‐Theme 2.1. Navigating Peer Relationships and Emotional Safety

Peer relationships within inpatient settings were described as complex and requiring careful navigation. When positive, peer connections were highly valued by service users, offering mutual understanding, empathy, and a sense of belonging that many felt was difficult to find elsewhere. These relationships provided validation through shared experience and, at times, supported emotional resilience and recovery.

However, peer interactions were not always experienced as supportive. Some service users described the ward environment as intimidating, particularly when others' aggression or complex needs created a sense of threat. In these circumstances, individuals reported focusing on survival rather than recovery, carefully managing interactions to avoid conflict or distress.

My dad used to get mad and say, why do you hit people for? You'll be there for a long time. I said, dad, you don't understand. You have to stand up for yourself. If I don't stand up, people will walk all over me […] I used to keep myself to myself. And when [staff member] said to me, that's the best way to get out of this place, is you got to keep yourself to yourself and get out.

(Service user interview #1, Community).

Across these accounts, staff were consistently viewed as essential in providing protection, emotional containment, and guidance, supporting service users to regulate emotions and maintain safety. The dual nature of peer relationships, simultaneously offering connection and posing risk, highlighted the importance of active staff oversight and boundary‐setting to facilitate recovery rather than hinder it.

#### 3.2.2 Sub‐Theme 2.2. Engagement With Therapeutic Interventions and Meaningful Activity

Active engagement with therapeutic interventions and meaningful activities was described as a central mechanism for sustaining progress and readiness for discharge. Service users described how understanding their treatment supported coping and a sense of empowerment and control. Medication was described by some as promoting emotional stability and enabling greater engagement in recovery and preparation for discharge.

Because me and [professional's name] did it, it were [professional's name] who did it with me. And I thanked [professional's name], I couldn't thank her enough.

(Service user interview #4, Community).

Medication is very important… The medication I have [the Doctor] put me on. It's a good medication. Keeps me stable. Keeps me calm. I have a good sleep. I feel myself.

(Service user interview #1, Community).

Psychological therapies were similarly described as pivotal to progression, with some service users explicitly linking engagement to discharge outcomes:

…if I hadn't have done what I did, which was CBT, I wouldn't have been discharged now.

(Service user interview #4, Community).

Staff reinforced the importance of early and sustained engagement, emphasising that therapeutic and meaningful activities should begin at admission and continue throughout the inpatient stay. These activities extended beyond formal therapy to include skill development, independence‐focused tasks, and community engagement, all of which were viewed as supporting confidence, autonomy, and continuity across the pathway.

…we need to focus right from the start and get the patients really engaged in meaningful things for them….

(Staff focus group #5, P3)

Collectively, these accounts illustrate engagement not as a discrete intervention, but as an ongoing process that maintained momentum and reinforced individuals' sense of agency and progression.

#### 3.2.3 Sub‐Theme 2.3. Communication, Collaboration, and Shared Objectives

Participants across groups highlighted the importance of clear communication and collaborative planning in maintaining coherence across the care trajectory. Clinical staff described the value of co‐produced, personalised discharge planning, with shared objectives providing a sense of direction and collective responsibility.

Feeling like [there is] a shared objective and the service users being people who own those objectives and goals too.

(Staff focus group #4, P1)

Service users and carers similarly emphasised the importance of understanding expectations and being actively involved in planning processes. Where communication was transparent and consistent, individuals described feeling more prepared and confident. Many service users described staff as instrumental in keeping them informed, listening to their concerns, and preparing them for their community placements during their discharge pathway. This preparation often involved gradually reducing levels of support and restrictions to encourage independence, as well as arranging community visits or overnight stays to build confidence and familiarity with placements. Such support was particularly valued by service users during the discharge process as it helped to reduce uncertainty and frustration while reinforcing trust and readiness for transition.

It was easier because I actually knew when I was going […] they actually told me when I was going, so I actually knew the date when I was going.

(Service user interview #2, community).

Conversely, unclear or changing messages contributed to anxiety, disengagement, and mistrust. Staff noted that external pressures, including legal restrictions, commissioning processes, and resource limitations, often complicated efforts to maintain clarity and continuity. These constraints sometimes resulted in delays or mixed messages that undermined shared understanding, even when clinical teams were working collaboratively.

Trying to discharge someone from another hospital to [location] from [location]. There's supposed to be a three‐month handover period but this doctor says they don't want that, they do it very differently. It's gone to six months where he's on Section 17 transition and still not willing to take [the risk] […] because of the forensic issues.

(Staff focus group #1, P4)

One staff participant illustrated how incomplete actions could defer progress between statutory aftercare reviews:

It's small actions, isn't it, that need to be done and they're not completed and these actions could be done and then the next process it's like “oh, we didn't realise”. Then another four weeks the next 117 [Section 117 aftercare review] and then it's something else and another two months have gone by for a simple action, just to complete the form.

(Staff focus group #1, P3).

These findings suggest that maintaining continuity across the care trajectory requires not only therapeutic input, but also sustained coordination, transparency, and shared ownership of goals across disciplines and agencies.

Taken together, Theme 2 highlights continuity as an actively maintained process rather than a static feature of care. Peer dynamics, engagement with interventions, and collaborative planning emerged as interdependent mechanisms shaping whether individuals experienced coherence or fragmentation during their inpatient journey. Where emotional safety was supported, engagement sustained, and objectives shared, participants described momentum towards discharge. Where these processes faltered, continuity weakened, increasing the risk of stalled progress and reinforcing cyclical patterns of admission and delay.

### 3.3 Theme 3. Sustaining Community Living and Preventing Re‐Entry Into Institutional Care

For participants across all groups, discharge was not experienced as the endpoint of the forensic care pathway, but as a transition into a new and often fragile phase of recovery. Sustaining community living depended on the extent to which continuity of support, identity, and relationships could be maintained beyond hospital settings. Where continuity broke down, participants described heightened anxiety, risk of relapse, and, for some, re‐entry into institutional care.

#### 3.3.1 Sub‐Theme 3.1. Identity, Freedom, and Belonging in the Community

Service users described discharge as both a highly valued and emotionally complex transition. Returning to the community was often associated with a renewed sense of freedom, autonomy, and identity, particularly after long periods in secure settings. Being able to make everyday choices and imagine a future outside hospital was described as central to recovery.

I'm independent now, but I was proper here and I did need support from staff when I was in there, but I'm much myself now and I feel like I've done well to get out of that place.

(Service user interview #1, community).

However, this transition was also characterised by uncertainty and vulnerability. Some service users described ongoing restrictions, supervision, or rules that felt disconnected from their current circumstances, creating confusion about what ‘being discharged’ actually meant.

I don't like staff with me all the time, because I can do cleaning by myself, cook by myself. Why do I need staff with me 24 hours?

(Service user interview #1, community).

Carers similarly, described the emotional complexity of this phase, expressing relief at their relative's discharge alongside concerns about whether adequate support was in place to sustain independence and safety.

We're very happy with how it's worked out with her. And she's reasonably independent […] Even when she goes shopping and does her banking and all that she has somebody with her to help her. But it's just fantastic.

(Carers couple interview #2, P1)

These accounts suggest that sustaining community living involves not only physical relocation, but ongoing support for identity reconstruction, autonomy, and belonging.

#### 3.3.2 Sub‐Theme 3.2. Continuity of Support and Managing Vulnerability Post‐Discharge

Continuity of care following discharge emerged as a critical factor in preventing relapse and re‐admission. While service users appreciated their freedom and independence, they equally emphasised the importance of having access to a key familiar/trusted professional, particularly during times when community placements felt unsafe or when personal crises arose. Where support was perceived as fragmented or withdrawn too quickly, individuals described feeling isolated and overwhelmed.

One service user shared that they were readmitted to hospital following a mental health crisis triggered by the death of a close relative, believing that earlier support could have prevented this relapse. Another service user described uncertainty about who to contact when they felt unsafe in their placement. The same individual disclosed that they were later seriously injured in the very placement they had feared. Carers echoed these concerns, whilst also highlighting the importance of continuity in staffing so that individuals clearly understood who to contact when in need. Both service users and carers also identified practical areas where ongoing support was essential, such as managing finances and utility bills. Collectively, participants described how the diverse and evolving needs of service users are not always adequately met following discharge, leaving some vulnerable to instability and potential readmission.

So, another advice that I would give, if you are in community services and you're having problems with your service, make sure you get in touch with your social worker and stuff. They can sort it out and just make sure you don't get left on your own like I did.

(Service user interview #10, inpatient)

This tension was also evident in service users' accounts of continuous 24/7 staffing: support could provide safety and stability, while support experienced as excessive could restrict autonomy and confidence.

Staff echoed these concerns, describing the challenges of ensuring continuity when individuals transitioned between services with different thresholds, resources, and risk tolerances. They noted that community services were not always equipped to manage forensic risk, increasing the likelihood of breakdown and re‐admission.

The issue is very much around securing accommodation. It's been a real minefield […] to try and get him suitable accommodation […] he's ready for discharge but getting the right services in place for him has been a challenge.

(Staff focus group #2, P1)

Other staff attributed gaps in support to limited resources within community settings.

It's all obviously resource dependent. You have to go through funding applications, etcetera.

(Staff focus group #2, P2)

Carers described being left to manage risk and uncertainty with limited guidance, particularly during the early stages of discharge, reinforcing the importance of structured aftercare and clear points of contact.

#### 3.3.3 Sub‐Theme 3.3. Redefining ‘Successful Discharge’ and Learning From Setbacks

Across stakeholder groups, there was recognition that traditional definitions of ‘successful discharge’, often framed as the physical move out of hospital, failed to capture the lived realities of recovery and risk. Stability, quality of life, autonomy and meaningful relationships were identified as important indicators of success. Staff accounts also suggested that setbacks and re‐admissions could provide opportunities for reviewing support arrangements and adapting future care, rather than being understood solely as individual failure.

A flexible and learning‐oriented response to setbacks was therefore considered important for supporting renewed engagement and reducing the likelihood that re‐admission would lead to another prolonged inpatient stay.

Taken together, Theme 3 illustrates participants' accounts of community living as dependent on continuity of appropriate support and relationships beyond discharge. Where this continuity was achieved, participants described increased confidence, autonomy, and stability. Where it was disrupted, individuals became vulnerable to crisis and re‐admission. These findings underscore the importance of viewing discharge not as an endpoint, but as a transition within an ongoing care trajectory that must be actively supported to prevent cyclical patterns of admission and delay.

## 4 Discussion

This study examined how adults with intellectual disabilities and forensic histories experience discharge and transition from secure hospital settings, drawing on the perspectives of service users, family members, and multidisciplinary professionals, and validating findings through co‐production workshops with a large and diverse workforce. The findings suggest that participants experienced discharge not as a linear or time‐limited event, but as part of an ongoing care trajectory characterised by movement into, through and out of services. Across stakeholder groups, continuity of relationships, planning and support was consistently described as influencing whether individuals experienced progression through the pathway or became vulnerable to prolonged or repeated cycles/episodes of institutional care.

### 4.1 Discharge as a Cyclical Process Rather Than a Linear Endpoint

A key contribution of this study is the empirical challenge it poses to linear models of forensic care that conceptualise admission, treatment, and discharge as discrete and sequential stages. Participants consistently described pathways as unstable and recursive, with delayed discharge, community breakdowns, and readmissions forming part of a repeating cycle rather than isolated failures. These findings extend existing evidence documenting long lengths of stay and delayed discharge among people with intellectual disabilities in forensic settings (Hatton 2016; Ince et al. 2022; Taylor et al. 2017) by illuminating how these systemic patterns are experienced and interpreted by those most closely affected.

The findings align with current policy concerns in England regarding inappropriate long‐term detention and the need to reduce reliance on inpatient care for people with intellectual disabilities (NHS England 2019, 2024). The Mental Health Act 2025 provides for future changes to the detention criteria for people with intellectual disabilities and autistic people under Part II of the Mental Health Act 1983. These provisions have not yet been brought into force. At present, a person with a learning disability may still fall within the Section 3 detention framework where the disability is associated with abnormally aggressive or seriously irresponsible conduct; evidence of a separate co‐occurring mental illness is not required. Once commenced, the 2025 Act will prevent detention for treatment under Section 3 on the basis of intellectual disability or autism alone and will require a qualifying psychiatric disorder. Implementation is being prepared through ongoing work by NHS England and the Department of Health and Social Care, including development of appropriate community infrastructure (NHS England 2025b). Importantly, these changes concern Part II and do not alter the equivalent detention framework for Part III patients involved in criminal proceedings or under sentence. For the forensic population considered in this study, timely progression and discharge therefore continue to depend on coordinated clinical, legal, commissioning, and community processes.

Participants' accounts also highlighted the influence of restriction orders and Ministry of Justice oversight on discharge planning. Although these safeguards are intended to support public protection, requirements for external authorisation may introduce additional complexity, uncertainty and delay, particularly where legal processes, clinical assessments and community arrangements are not well coordinated. Early and transparent communication about restrictions, authorisation requirements and their implications for leave and discharge is therefore important for service users, families and professionals across the pathway.

### 4.2 Continuity as the Organising Principle of Effective Forensic Pathways

Across all three themes, continuity emerged as the organising principle underpinning effective discharge and sustained community living. Continuity was understood relationally (through stable, trusting relationships), narratively (through shared understanding of recovery goals and risk), and practically (through coordinated planning and support across inpatient and community services). Where continuity was maintained, participants described momentum, confidence, and progression; where it was disrupted, individuals became vulnerable to disengagement, relapse, and re‐admission.

These findings build directly on a recent scoping review of models of care for people with intellectual disabilities and forensic histories (Haines‐Delmont et al. 2025), which identified continuity, early discharge planning, and integrated community pathways as common features of promising approaches internationally, while highlighting the lack of robust empirical guidance on how such principles are operationalised in practice. The present study addresses this gap by providing detailed, multi‐stakeholder insight into how continuity is enacted, undermined, and restored within real‐world forensic systems.

This emphasis on continuity also resonates with international frameworks, including the World Health Organisation's call for a shift towards community‐based, recovery‐oriented mental health care (World Health Organization 2025a) and the United Nations Convention on the Rights of Persons with Disabilities, which emphasises autonomy, inclusion, and the right to live independently in the community (United Nations 2006). The findings suggest that continuity across forensic pathways is not only a clinical or organisational concern, but also a matter of rights and social justice.

### 4.3 Structuring Discharge: Clarity, Consistency, and Shared Accountability

Participants across groups highlighted the importance of structured and transparent discharge processes that provide clarity and consistency for service users, carers, and professionals. Uncertainty, shifting expectations, and opaque decision‐making were described as major sources of anxiety and disengagement, undermining confidence and readiness for transition.

During interviews, focus groups, and co‐design workshops, participants repeatedly referred to established discharge planning approaches, for example the 12‐Point Discharge Plan (NHS England 2023a) that emphasise early planning, clear milestones, shared responsibility, and coordination across legal, clinical, housing, and commissioning domains was often described as ‘good practice’. While these approaches were not presented as novel or proprietary frameworks, they were consistently valued as practical tools for supporting continuity across the care trajectory.

The participant‐identified principles summarised in Table 1 illustrate how existing good practice can be clarified and embedded to support continuity across the forensic care trajectory. Although these principles do not prescribe a single model of implementation, they provide a practical framework through which services and commissioners can review existing pathways and identify locally appropriate actions to strengthen continuity. The participant‐identified principles summarised in Table 1 illustrate how existing good practice can be clarified and embedded to support continuity across the forensic care trajectory. While the principles outlined in Table 1 do not prescribe a single model of implementation, they provide a shared framework through which services and commissioners can review existing pathways and identify locally appropriate actions to improve continuity across the forensic care trajectory.

**TABLE 1:** Participant‐identified principles for improving forensic discharge pathways for adults with intellectual disabilities.

Domain	Participant‐identified good practice principles	Implications for services and commissioners
Pathway design	Discharge planning begins at admission and is oriented towards return to the community	Pathways should be community‐anchored, not security‐level driven
Continuity of care	Consistent relationships across inpatient and community services	Invest in workforce stability and relational continuity
Shared understanding	Clear, transparent communication of goals, risks, and expectations	Reduce uncertainty and disengagement for service users and carers
Therapeutic engagement	Early and sustained access to meaningful activity and intervention	Maintain momentum and readiness throughout admission
Risk management	Positive‐risk approaches shared across services	Avoid risk‐averse practices that delay discharge
Legal processes	Early planning around restrictions and legal thresholds	Minimise late‐stage delays linked to legal uncertainty
Housing	Suitable accommodation identified early	Prevent discharge delays driven by housing shortages
Commissioning	Alignment between clinical goals and commissioning decisions	Reduce system‐level fragmentation
Aftercare	Structured post‐discharge follow‐up and support	Reduce risk of relapse and re‐admission
Learning from setbacks	Readmissions framed as learning opportunities	Avoid punitive or blame‐based responses
Carer involvement	Inclusion of families/carers where appropriate	Strengthen support networks and continuity
System accountability	Shared ownership across inpatient and community teams	Move from service‐led to person‐centred pathways

*Note:* These principles reflect participant‐identified good practice and are intended to inform local service development rather than prescribe a single model of implementation.

### 4.4 Implications for Policy, Practice, and Commissioning

The findings suggest several implications for policy and practice. First, forensic pathways for people with intellectual disabilities should be explicitly designed around continuity across the care trajectory rather than linear progression through institutional stages. Second, discharge planning should be conceptualised as a longitudinal process, beginning early and extending beyond physical discharge, with shared ownership across inpatient and community services. Third, commissioners and service providers should prioritise investment in community capacity, workforce stability, and integrated models of support capable of managing forensic risk without defaulting to re‐institutionalisation.

The validation of findings through co‐production workshops involving over 90 multidisciplinary professionals strengthens the credibility and feasibility of these implications. Participants not only recognised the findings as reflective of their experience but also contributed to the identification of realistic, system‐level actions to improve discharge pathways within existing constraints.

### 4.5 Strengths and Limitations

This study is strengthened by its inclusion of multiple stakeholder perspectives across inpatient and community forensic intellectual disability services, including service users, family members and a professionally diverse multidisciplinary workforce. The subsequent validation of findings through two large co‐production workshops strengthened the credibility, resonance and practical relevance of the analysis by ensuring that interpretations reflected the experiences of those delivering and receiving forensic care. Although the study included a professionally diverse multidisciplinary workforce alongside service users and family members, it did not include representatives from the Ministry of Justice, commissioners, housing providers or other agencies involved in discharge decision‐making. Future research should explore how these perspectives interact with those of service users, families and clinical teams to influence progression through forensic pathways.

The study also has several limitations. Data were collected within a single NHS trust, which may limit transferability to other service contexts. In addition, although the study focused specifically on adults whose primary diagnosis was an intellectual disability, autism was not examined as a distinct analytic characteristic. Future research should explore whether similar continuity‐focused principles apply across autistic forensic populations and people with co‐occurring intellectual disabilities and autism. Finally, the absence of longitudinal outcome data means that future implementation research is needed to evaluate the impact of continuity‐focused discharge practices over time.

## 5 Conclusion

Improving the quality, coordination and sustainability of discharge from forensic intellectual disabilities services requires a shift away from linear, service‐led pathways towards continuity‐focused care trajectories that begin and end in community living. By foregrounding service user experiences and integrating them with the perspectives of family members and multidisciplinary professionals, this study provides empirically grounded evidence to inform policy, commissioning and clinical practice. Rather than proposing a new model of care, the findings identify practical principles that can strengthen existing forensic pathways, support safe and sustainable community living and reduce avoidable cycles of readmission.

## Author Contributions

Conceptualisation: A.H.‐D. Methodology: A.H.‐D., M.T. Data collection: S.A., S.R., M.T., A.H.‐D.; Qualitative analysis: A.H.‐D., K.F., F.M., S.A., S.R., M.T. Writing: A.H.‐D., K.F. Manuscript review, editing and approval: S.A., S.R., F.M., M.T. Supervision: A.H.‐D., M.T. Funding acquisition: A.H.‐D.

## Funding

This work was supported by Mersey Care NHS Foundation Trust.

## Ethics Statement

The authors assert that all procedures contributing to this work comply with the ethical standards of the relevant national and institutional committees on human experimentation and with the Helsinki Declaration of 1975, as revised in 2013. All procedures involving human subjects/patients were approved by Manchester Metropolitan University [EthOS reference 49315], the HRA and HCRW [Yorkshire & The Humber—Leeds West Research Ethics Committee, IRAS ID: 324747].

## Consent

All persons gave their informed consent prior to their inclusion in the study.

## Conflicts of Interest

The authors declare no conflicts of interest.

## Data Availability

The datasets generated and/or analysed during the current study are not publicly available due to the sensitive nature of the investigation and to protect the confidentiality of participants, especially the individuals with lived experience, but are available from the corresponding author on reasonable request.
